# A stab wound to the axilla illustrating the importance of brachial plexus anatomy in an emergency context: a case report

**DOI:** 10.1186/s13256-016-1162-6

**Published:** 2017-01-04

**Authors:** Diogo Casal, Teresa Cunha, Diogo Pais, Inês Iria, Maria Angélica-Almeida, Gerardo Millan, José Videira-Castro, João Goyri-O’Neill

**Affiliations:** 1 Plastic and Reconstructive Surgery Department and Burn Unit, Centro Hospitalar de Lisboa Central, Lisbon, Portugal; 2 Anatomy Department, NOVA Medical School, Universidade NOVA de Lisboa, Campo dos Mártires da Pátria, 130, 1169-056 Lisbon, Portugal; 3 UCIBIO, Life Sciences Department, Faculty of Sciences and Technology, Universidade NOVA de Lisboa, Caparica, Portugal; 4 CEDOC, NOVA Medical School, Universidade NOVA de Lisboa, Lisbon, Portugal

**Keywords:** Brachial plexus, Brachial plexus injuries, Brachial plexus anatomy, Wounds and injuries, Peripheral nervous system, Neurological examination, Nerve repair, Case report

## Abstract

**Background:**

Although open injuries involving the brachial plexus are relatively uncommon, they can lead to permanent disability and even be life threatening if accompanied by vascular damage. We present a case report of a brachial plexus injury in which the urgency of the situation precluded the use of any ancillary diagnostic examinations and forced a rapid clinical assessment.

**Case presentation:**

We report a case of a Portuguese man who had a stabbing injury at the base of his left axilla. On observation in our emergency room an acute venous type of bleeding was present at the wound site and, as a result of refractory hypotension after initial management with fluids administered intravenously, he was immediately carried to our operating room. During the course of transportation, we observed that he presented hypoesthesia of the medial aspect of his arm and forearm, as well as of the ulnar side of his hand and of the palmar aspect of the last three digits and of the dorsal aspect of the last two digits. Moreover, he was not able to actively flex the joints of his middle, ring, and small fingers or to adduct or abduct all fingers. Exclusively relying on our anatomical knowledge of the axillary region, the site of the stabbing wound, and the physical neurologic examination, we were able to unequivocally pinpoint the place of the injury between the anterior division of the lower trunk of his brachial plexus and the proximal portion of the following nerves: ulnar, medial cutaneous of his arm and forearm, and the medial aspect of his median nerve. Surgery revealed a longitudinal laceration of the posterior aspect of his axillary vein, and confirmed a complete section of his ulnar nerve, his medial brachial and antebrachial cutaneous nerves, and an incomplete section of the ulnar aspect of his median nerve. All structures were repaired microsurgically. Three years after the surgery he showed a good functional outcome.

**Conclusions:**

We believe that this case report illustrates the relevance of a sound anatomical knowledge of the brachial plexus in an emergency setting.

## Background

Although open injuries involving the brachial plexus (BP) are relatively uncommon nowadays, not only can they lead to permanent severe limb dysfunction, but they also might be life threatening, since many of these injuries are accompanied by vascular damage and sometimes even by lung injury [1–7]. In such emergency situations, immediate surgical exploration is necessary and there is consensus for simultaneous vascular and nerve repair [4, 8, 9]. Immediate nerve repair also minimizes the need for nerve grafts, flaps, or nerve reconstruction conduits [5].

Therefore, the only opportunity to assess and evaluate the patient is often during the transfer from the emergency department to the operating room. In these circumstances, clinical evaluation might be the only diagnostic tool and therefore plays a pivotal role in early diagnosis and surgical planning [4, 8, 9]. In fact, a summary medical history and a directed physical examination are in most cases sufficient to identify the level of injury, the nerves involved, and the severity of injury [8, 9]. However, it should be noted that in many cases of open wounds associated with major vascular bleeding, patients are too unstable for even a summary neurological examination to be made prior to transport to the operating room [10]. In fact, frequently patients are carried to the emergency room already under sedation and ventilated [8–10]. Depending on the severity and degree of vascular involvement, the urgency of these situations may even preclude the use of any ancillary diagnostic methods and force a rapid clinical assessment based on a sound knowledge of BP anatomy [10].

Even though there have been reports of BP lesions since at least the eighth century BC in Homer’s *Iliad* [11], even today the complexity, multiplicity, and potential anatomical variations of these structures make the study of the topographic anatomy of the axilla and that of the cervical-thoracic outlet a difficult subject for health professionals in general [12–14].

## Case presentation

A 40-year-old right-handed Portuguese man was brought to our Emergency Department 10 minutes after sustaining a stab wound to the base of his left axillary region after being mugged. His past medical history was unremarkable.

On observation, a profuse acute venous type bleeding was present at the wound site. The wound was located in the middle of his left axilla. It measured approximately 3 cm in length and was oriented in an anterior–posterior axis. A compressive dressing was applied at the entry point of the stab wound. As a result of refractory hypotension after initial management with vigorous fluidotherapy, he was immediately carried to our operating theatre.

**Fig. 1:**
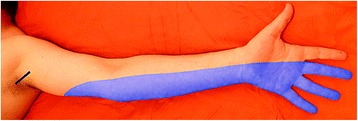
Picture illustrating the area of hypoesthesia presented by the patient at admission. The shading area corresponds to the patient's area of hypoesthesia. The black line represents the site of the stabbing wound

During the course of transportation, it was possible to clinically assess his left upper limb in a summary fashion. Pinprick and light touch sensory examination revealed hypoesthesia of the medial aspect of his arm and forearm from the axillary crease to the palmar wrist crease, as well as of the ulnar side of his hand and of the palmar aspect of the last three digits and of the dorsal aspect of the last two digits (Fig. 1). All other areas of his left upper limb showed a normal sensory response.

**Fig. 2:**
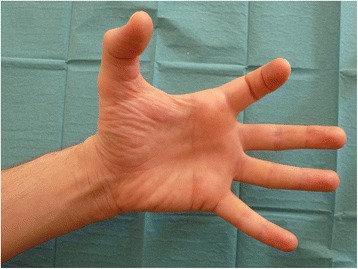
Photograph illustrating the motor deficit presented by the patient at admission. The patient was not able to flex joints of the middle, ring and little fingers of the left hand

A motor examination revealed that he was not able to actively flex the joints of his middle, ring, and little fingers nor to adduct or abduct any of the fingers of his left hand (Fig. 2). Moreover, he was not able to adduct his wrist. The remaining motor examination of his left upper limb showed no deficits.

**Fig. 3:**
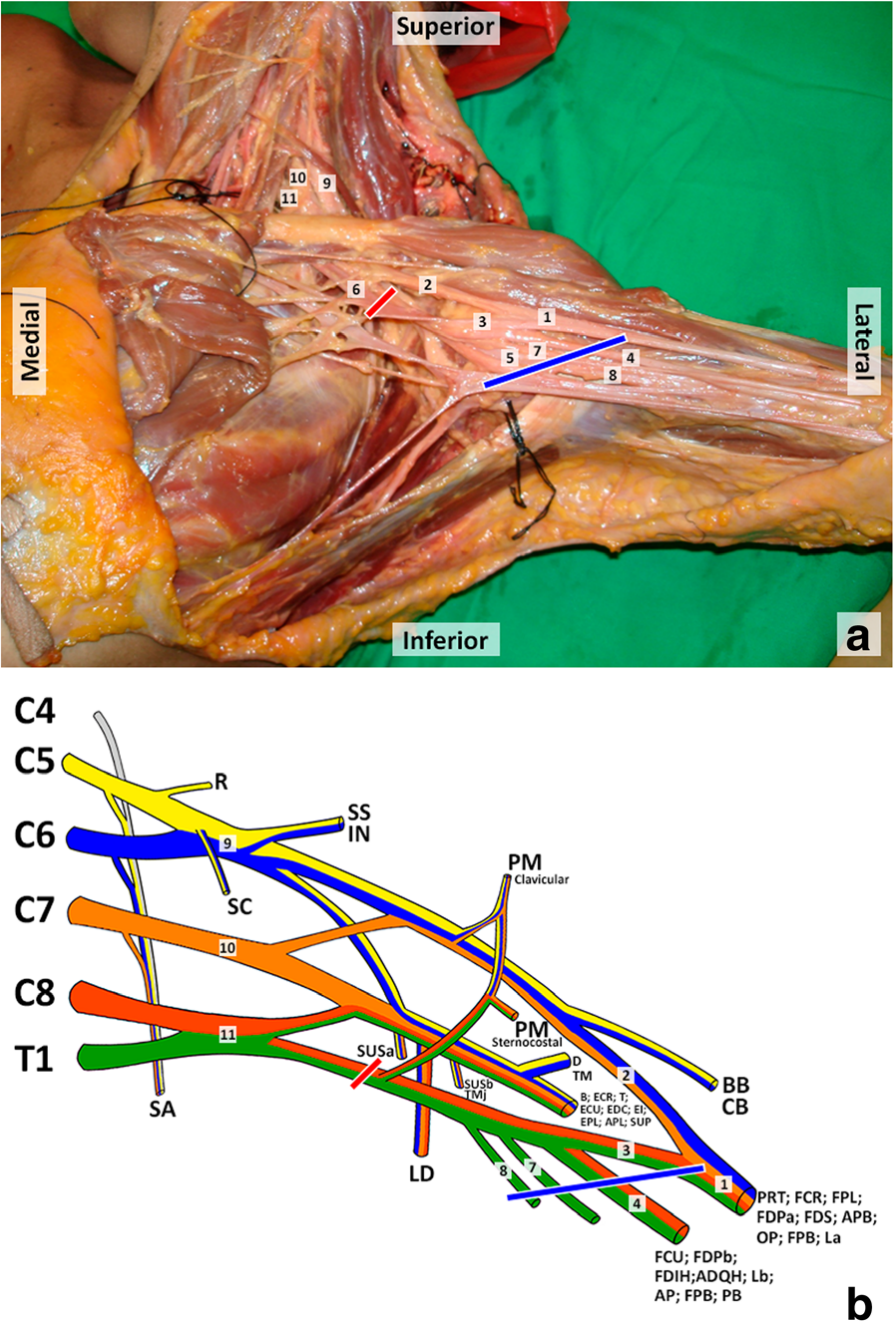
Brachial plexus composition, neighboring structures and territory. The most proximal and distal places of the possible lesion site according to the symptomatology presented by the patient are heralded by the red and blue lines. **a**. Photograph of a cadaveric dissection of the left axillary region showing the brachial plexus, its terminal branches and their neighboring structures. 1, median nerve; 2, median nerve root side; 3, medial root of the median nerve; 4, ulnar nerve; 5, axillary artery; 6, axillary vein; 7, medial cutaneous nerve of the arm; 8, medial cutaneous nerve of the forearm; 9, upper trunk of the brachial plexus; 10, middle trunk of the brachial plexus; 11, lower trunk of the brachial plexus. **b**. Schematic drawing of the brachial plexus, roots, trunks, divisions, cords, terminal branches, and the muscles (m.) they innervate. SA, serratus anterior m.; SC, subclavius m.; R, rhomboids m.; SS, supraspinatus m.; IN, infraspinatus m.; PM, pectoralis major m.; SUSa, subscapularis m. (upper half); LD, latissimus dorsi m.; SUSb, subscapularis m. (lower half); TMj, teres major m.; D, deltoid m.; TM, teres minor m.; B, brachioradialis m.; ECR, extensor carpi radialis m.; T, triceps m.; ECU, extensor carpi ulnaris m.; EDC, extensor digitorum communis m.; EI, extensor indicis proprius m.; EPL, extensor policis longus m.; APL, abductor pollicis longus m.; SUP, supinator m.; BB, biceps brachii m.; CB, coracobrachialis m.; PRT, pronator teres m.; FCR, flexor carpi radialis m.; FPL, flexor pollicis longus m.; FDPa, flexor digitorum profundus m. [bellies for the index and middle finger]; FDS, flexor digitorum superficialis m.; APB, abductor polllicis brevis m.; OP, opponens pollicis m.; FPB, flexor pollicis brevis m.; La, first and second lumbricals m.; FCU, flexor carpi ulnaris m.; FDPb, Flexor digitorum profundus m. [bellies for the ring and small fingers]; FDIH, first dorsal interosseous m.; ADQH, abductor digiti quinti m.; Lb, third and fourth lumbricals m.; AP, adductor pollicis m.; FPB, flexor pollicis brevis m. (deep head); PB, palmaris brevis m

**Fig. 4:**
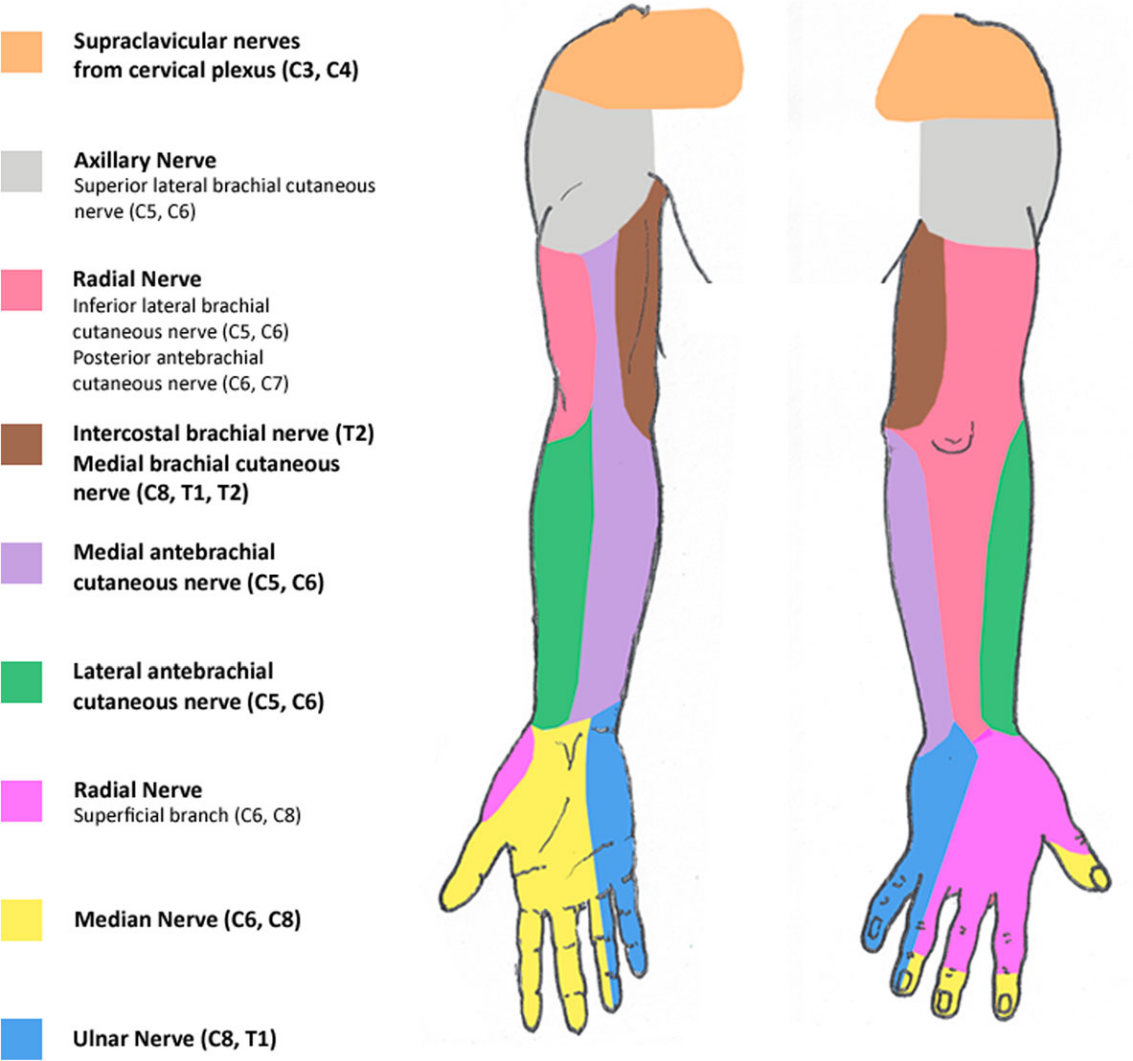
Schematic representation of the sensory innervation of the upper limb. The skin territories of the branches of the brachial plexus are shown

The clinical presentation enabled us to promptly locate the nerve injury between the anterior division of the lower trunk of his BP and the proximal portion of his following nerves: ulnar, medial cutaneous of his arm and forearm, and the medial aspect of his median nerve (Figs. 3 and 4).

**Fig. 5:**
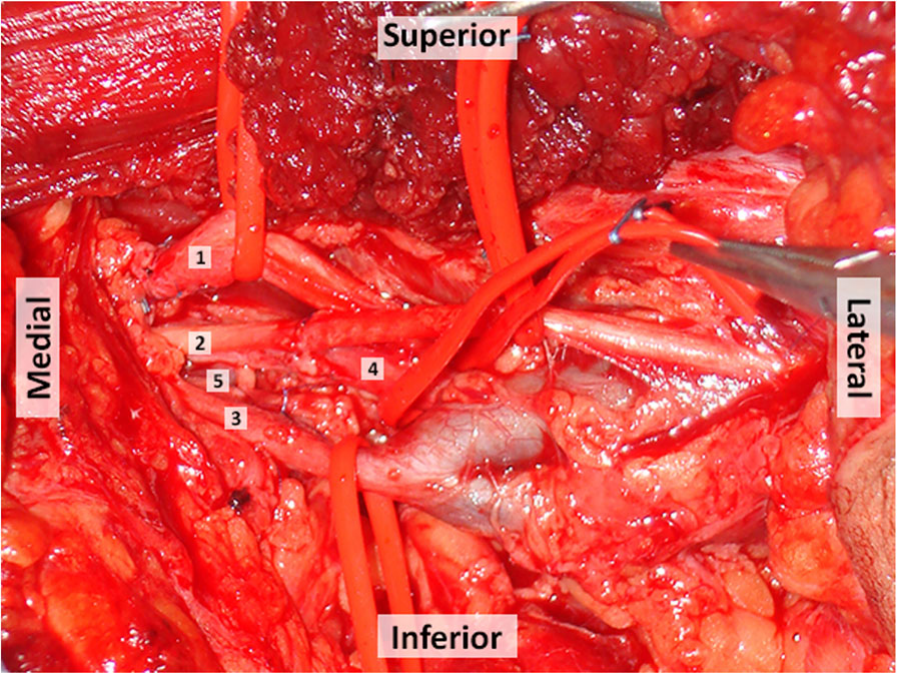
Photograph of the patient’s left axilla showing the intraoperative view of the axillary wound after control of bleeding and nerve repair. Longitudinal section of the posterior side of the axillary vein along with complete section of the ulnar, medial brachial cutaneous and medial antebrachial cutaneous nerves as well as partial section of the median nerve were found. 1, median nerve; 2, ulnar nerve; 3, axillary vein; 4, medial brachial cutaneous nerve; 5, medial antebrachial cutaneous nerve

Surgical exploration revealed a longitudinal laceration of the posterior aspect of his axillary vein, as well as a complete section of his ulnar nerve, his medial brachial and antebrachial cutaneous nerves, and an incomplete section of the ulnar aspect of his median nerve (Fig. 5). A surgical approach was made under surgical loupes’ magnification. It began with vessel repair using an interrupted 8/0 Nylon suture, followed by direct end-to-end repair of the severed nerves using 8/0 Nylon simple stitches. Fibrin glue was applied around the repaired nerves.

His postoperative period was uneventful. He started an intensive physiotherapy program after hospital discharge, which occurred 3 days after surgery. The physiotherapy was aimed at maintaining joint mobility and at strengthening the paralyzed muscles, as reinnervation occurred. Physiotherapy was performed daily for the first year after surgery and three times a week for the following year. In the postoperative period, he also started swimming following the attending physician’s advice.

**Fig. 6:**
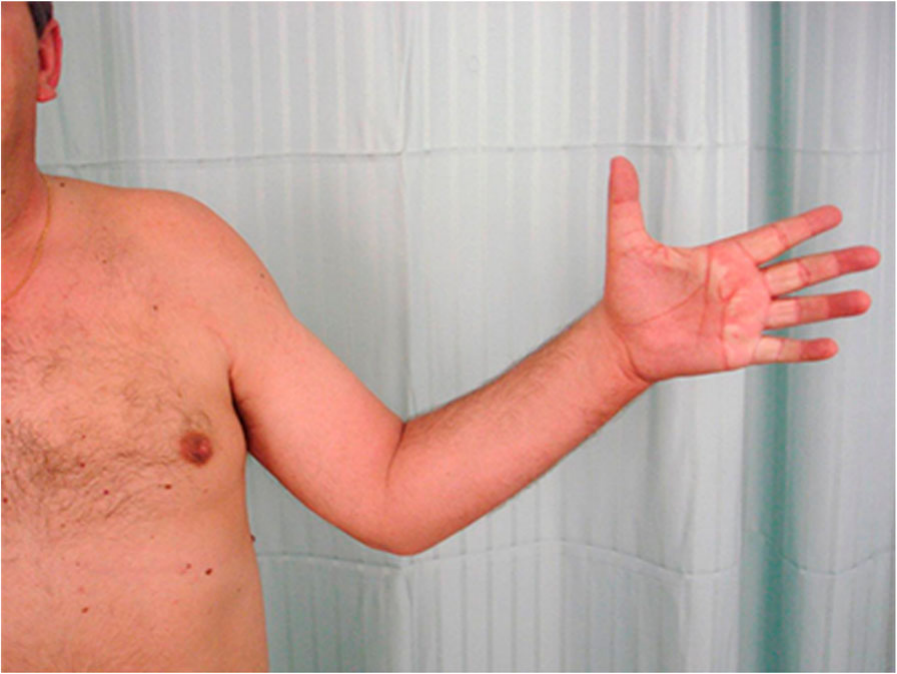
Photograph of the patient's left upper limb three years after surgery. There is evidence of slight atrophy of the muscles innervated by the ulnar and median nerves, but its overall function is good. There is a slight limitation in the maximal extension of the metacarpal-phalangeal joint of the fifth finger

**Fig. 7:**
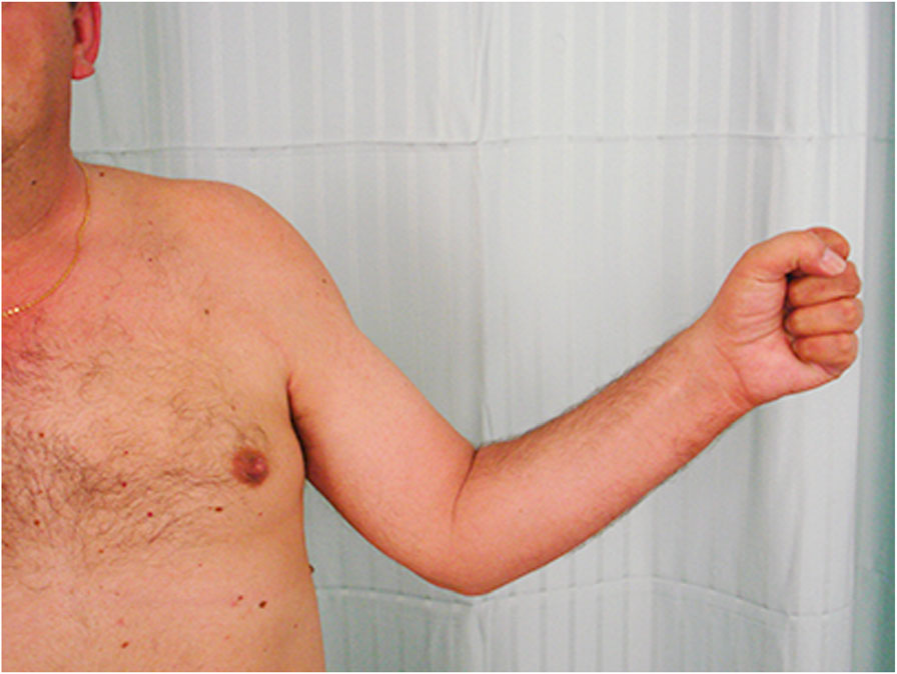
Photograph of the patient's left upper limb three years after surgery. The patient was able to fully flex all fingers, although with less strength than in the contralateral side

**Fig. 8:**
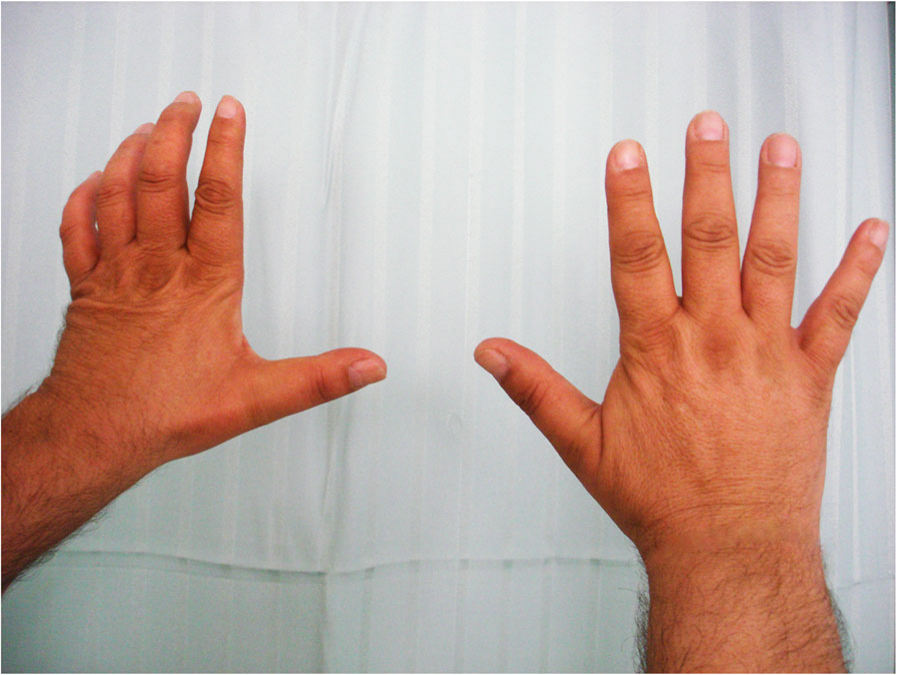
Photograph of the dorsum of the hands one year after surgery, showing slight atrophy of the muscles innervated by the ulnar and median nerve, as well as a mild ulnar claw

One year after surgery he resumed his employment. Three years after surgery, even though there was a slight atrophy of the intrinsic muscles of his hand, he presented a good overall function of his left upper limb (Figs. 6, 7, 8 and 9). At the last evaluation, 3 years after the accident, his motor function was M4 in all the previously paralyzed muscles according to the Medical Research Council Scale (muscle strength was reduced but muscle contraction could still move joints against resistance) [15]. Moreover, according to this scale [15], his sensory recovery was defined as S3 (return of superficial cutaneous pain and tactile sensibility without over-response) at the medial aspect of his arm and forearm, and as S2 (return of superficial cutaneous pain and some degree of tactile sensibility) at the ulnar side of his hand and at the palmar aspect of the last three digits and at the dorsal aspect of the last two digits.

This case report portrays a rare clinical situation in contemporary times: a major vascular lesion associated with a BP lesion in a conscious patient [16]. At present, this situation is rare because BP lesions are increasingly less frequent in most countries [16]. In addition, open BP injuries account for only a small percentage of all BP lesions [16–19]. In most cases of open BP wounds associated with major vascular bleeding, patients are too unstable for even a summary neurological examination to be made prior to transportation to the operating room. Most commonly, patients are carried to the emergency room already under sedation and ventilated. The patient presented in this case report was fortunate enough to have been close to the hospital at the time of the lesion. Therefore, despite the severe vascular damage, he did not yet have changes to his consciousness when he arrived at the trauma room. All these improbable events allowed a summary physical examination to be performed immediately before the emergency surgery. This in turn permitted a prompt diagnosis of the location of the nerve lesions, based solely on the physical findings and knowledge of anatomy.

In 2002, Dubuisson and Kline described 23 open BP injuries in 100 consecutive cases of BP lesions [20]. In 2003, from a total of 1019 patients with BP injuries, Kim *et al*. reported only 19% with open injuries, of which 7% involved lacerations and 12% were gunshot wounds [17]. Lacerations involving the BP may occur secondary to sharp instruments such as knives and glass, or from blunt trauma following animal bites or automobile accidents [5, 6, 17, 20–22]. These sources of injury most probably lead to neurotmesis (according to Seddon’s classification), which is the most severe type of injury to the peripheral nerves in which all the nerve layers are disrupted [7, 10].

Figure 3 illustrates the BP and the muscles innervated by each of its nerve branches. In most cases the convergence of the anterior rami of the spinal nerve roots from C5 to T1 forms the spinal nerve roots, the trunks, the divisions, the cords, and the terminal branches of the BP [23]. The terminal branches of the BP are responsible for most of the sensory, motor, and autonomic innervation of the upper limb (Fig. 4).

A classical aphorism in neurological diagnosis is to try to attribute all signs and symptoms to a single lesion whenever possible [24].

As can be seen in Fig. 3, the fact that our patient’s stab wound was at the base of his axilla, thereby inferior to his clavicle, suggested that the lesion was probably located at the level of the divisions, cords, or terminal branches of his BP [25].

The hypoesthesia of the medial aspect of his arm, forearm, and hand (Fig. 1) could be explained by: (a) a section of the anterior division of the lower trunk of his BP; (b) a complete section of the medial cord of his BP; (c) a complete section of his medial brachial and medial antebrachial cutaneous nerves, and his ulnar nerve, and a partial section of his median nerve (or medial root of his median nerve) [10, 12, 14, 26, 27].

Furthermore, paralysis of his flexor carpi ulnaris, medial part of his flexor digitorum profundus, third and fourth lumbricals, both palmar and dorsal interossei, adductor pollicis, abductor digiti minimi, flexor digiti minimi, and opponens digiti minimi muscles, indicates complete dysfunction of his entire ulnar nerve. The paralysis of the muscle bellies of his flexor digitorum superficialis and flexor digitorum profundus for his third finger suggests partial median nerve dysfunction. Once more, this motor dysfunction could be caused by: (a) a section of the anterior division of the lower trunk of his BP; (b) a complete section of the medial cord of his BP; (c) a complete section of his ulnar nerve, and a partial section of his median nerve (or medial root of his median nerve) [10, 12, 14, 26, 27].

A less likely cause of all these signs and symptoms could be either a lower trunk lesion or a lesion of the C8 and T1 roots of his BP. However, in either case, compromise of the nerves arising from his dorsal cord, namely of his radial nerve, causing motor dysfunction and sensory changes in the territory of this nerve at the level of his forearm and hand would be present. In addition, sharp injury to the T1 root seemed unlikely, as this root is very close to the T1 sympathetic ganglion, whose lesion would produce Horner’s syndrome ipsilaterally (meiosis, ptosis, enophthalmos, and facial anhydrosis) [10, 28].

With all these data taken into consideration, the region of the lesion of his BP could be safely pinpointed to the region between the anterior division of the lower trunk and the proximal portion of his ulnar, medial cutaneous nerves of his arm and forearm, and the medial aspect of his median nerve (Figs. 3 and 4). This in turn allowed a prompt planning of the surgical approach, and no doubt contributed to the good functional result observed 3 years after the surgery.

## Conclusions

We present an increasingly rare clinical situation in present times: a major vascular lesion associated with a BP lesion in a conscious patient. In this clinical case, knowledge of the clinical anatomy of this region allowed a prompt diagnosis of the location of the nerve lesions. This, in turn, permitted repair not only of the vascular damage that was jeopardizing the patient’s life, but also his severed nerves, which no doubt played a major role in saving his life and achieving the good functional results observed. We believe this case report eloquently demonstrates the clinical importance of a sound knowledge of the anatomy of the BP in an emergency clinical setting.

## Abbreviation

BP: Brachial plexus
